# *Toxoplasma gondii* seroprevalence in the Iranian blood donors: A systematic review and meta-analysis

**DOI:** 10.1016/j.heliyon.2024.e28013

**Published:** 2024-03-13

**Authors:** Masoud Foroutan, Hamidreza Majidiani, Soheil Hassanipour, Milad Badri

**Affiliations:** a Department of Basic Medical Sciences, Faculty of Medicine, Abadan University of Medical Sciences, Abadan, Iran; b Healthy Aging Research Centre, Neyshabur University of Medical Sciences, Neyshabur, Iran; c Department of Basic Medical Sciences, Neyshabur University of Medical Sciences, Neyshabur, Iran; d Gastrointestinal and Liver Diseases Research Center, Guilan University of Medical Sciences, Rasht, Iran; e Medical Microbiology Research Center, Qazvin University of Medical Sciences, Qazvin, Iran

**Keywords:** *Toxoplasma gondii*, Blood donors, Iran, Systematic review, Meta-analysis

## Abstract

**Background:**

The present systematic review and meta-analysis was done to assess the rate of *Toxoplasma gondii* (*T. gondii*) exposure among Iranian healthy blood donors.

**Methods:**

We searched four English (PubMed, Scopus, Web of Science, and Science Direct) and two Persian databases (Magiran and SID) as well as Google Scholar as a specialized article search engine using specific keywords for relevant papers from the inception up to November 30, 2023.

**Results:**

In total, 19 studies enrolling 8226 apparently healthy blood donors were examined regarding *T. gondii*-specific IgG and/or IgM antibodies, among which 2666 [32.9% (95% CI: 25.3%–41.6%)], 168 [1.4% (95% CI: 0.9%–2.1%)], and 83 [1.7% (95% CI: 1.3%–2.1%)] subjects were seropositive regarding IgG, IgM, and both IgG/IgM antibodies, respectively. Five risk factors were significantly associated with *T. gondii* seroprevalence, including gender (OR = 1.98; 95% CI: 1.52–2.58; *P* < 0.001), contact with cat (OR = 2.41; 95% CI: 1.70–3.41; *P* < 0.001), contact with soil (OR = 2.83; 95% CI: 1.07–7.45; *P* = 0.035), consuming raw/undercooked meat (OR = 1.95; 95% CI: 1.03–3.70; *P* = 0.039), and raw/unwashed vegetables (OR = 1.70; 95% CI: 1.25–2.31; *P* = 0.001).

**Conclusion:**

A moderate rate of *T. gondii* exposure was found in the Iranian blood donors, with the association of several risk factors, including gender, contact with cat, contact with soil, consumption of unwashed vegetables and/or undercooked meat. Still, more studies are recommended regarding *T. gondii* exposure among blood donors in Iran.

## 1 Introduction

*Toxoplasma gondii* (Apicomplexa, Sarcocystidae) remains a major global concern in one-third of the world population [1], with 33.8% and 35.9% pooled seroprevalence among pregnant women and immunocompromised patients, respectively [[2], [3], [4]]. Felids (definitive hosts) cause environmental contamination by dissemination of *T. gondii* oocysts [5]. Also, other sources of infection are undercooked meat and organ transplants containing tissue cysts (bradyzoites) as well as congenital transmission and blood transfusion *via* invasive of tachyzoites [1,6]. The infection in healthy individuals usually manifests with generalized signs such as sweating, malaise, myalgia, weight loss, fever and lymphadenopathy. The clinical disease, toxoplasmosis, is of particular concern in pregnant women with immune-modulated responses as well as those people having weakened immunity such as tissue graft recipients, cancer patients, and HIV-positive individuals. Reportedly, myocarditis, brain abscess, encephalitis and chorioretinitis are the major complications of toxoplasmosis in the at-risk individuals [7]. Moreover, *T. gondii* has been suggested to be positively-associated with several complications such as diabetes, obesity, cardiac disorders, depression, schizophrenia, and even brain tumors [[8], [9], [10], [11], [12]].

Blood transfusion is a therapeutic approach for a wide range of hematological malignancies and other diseases, including different types of anemia and thalassemia [13,14]. In this context, prevention of transfusion-transmitted infections (TTIs) is a critical checkpoint to improve the safety of blood products. Asymptomatic infections in the blood donors, which may persist upon blood processing and storage, would represent a serious risk of clinical disease to the recipients [15]. Previously, initial insights into the “transmission of toxoplasmosis by leukocyte transfusion” were elucidated by Siegel et al. (1970) [6], who reported the infection in two children with acute leukemia undergoing white blood cell (WBC) transfusion from a donor with chronic myelogenous leukemia. Based on a recent global meta-analysis study, the total seroprevalence of *T. gondii* among blood donors was estimated to be 33% (95% confidence interval (CI): 28%–39%) [16]; however, the global seroprevalence does not provide insight into the risk of transmission and only shows the pool from which seropositive individuals could be identified.

In Iran, *T. gondii* is one of the highly prevalent parasitic infections among different human populations [17,18]. The present systematic review and meta-analysis was performed to evaluate the rate of *T. gondii* exposure and associated risk factors among blood donors in Iran.

## 2 Methods

### 2.1 Study settings

A nation-wide systematic review and meta-analysis was designed and implemented to evaluate the seroprevalence of specific anti-*T. gondii* antibodies and associated risk factors among blood donor population in Iran. The reporting protocol was premised on the “Preferred Reporting Items for Systematic Reviews and Meta-Analysis (PRISMA)” guideline [19].

### 2.2 Search strategy

The systematic search process was performed through four English (PubMed, Scopus, Web of Science, and Science Direct) and two Persian databases (Magiran and SID) as well as Google Scholar as a specialized article search engine from the inception up to November 30, 2023 using the following keywords: (“*Toxoplasma*” OR “*Toxoplasma gondii*” OR “*T. gondii*” OR “toxoplasmosis”) AND (“prevalence” OR “seroprevalence” OR “epidemiology”) AND (“blood donor” OR “blood transfusion” OR “transfusion” OR “blood pack”) AND (“Iran” OR “Islamic republic of Iran”). To more cover relevant literature, the reference list of related papers was explored manually.

### 2.3 Eligibility criteria, study selection, and data extraction

The eligibility criteria for included studies were as follows: **1**) peer-reviewed cross-sectional studies reporting the rate of *T. gondii* exposure in healthy blood donors, **2**) examined *via* enzyme-linked immunosorbent assay (ELISA) method to detect specific anti-*T. gondii* antibodies in blood donors, **3**) papers published in both English and Persian languages, **4**) published online in international and national databases until November 30, 2023, and **5**) having available sample size and positive samples. It is noteworthy that case reports, reviews, letters, studies without evaluation of *T. gondii* antibodies, as well as studies with unclear/confusing information were totally excluded from the current systematic review and meta-analysis. In the next step, duplicated papers were removed, the title and abstract of the remaining papers were reviewed, and full-texts of qualified articles were obtained. The eligibility of the papers was evaluated independently by two researchers and likely contradictions were obviated through consultation with the leading investigator. In the following, a pre-designed Microsoft Excel Spreadsheet was used to extract the required information from included studies, including: the first author's last name, publication year, province, method, sample size, number of Immunoglobulin G (IgG) and/or Immunoglobulin M (IgM) positive samples, number of positive individuals based on gender, urban or rural residency, blood groups, Rh status, contact with cat and/or soil, history of blood transfusion, consumption of raw/undercooked meat, milk or egg, consumption of raw/unwashed vegetables, gardening or agriculture, and education level if available.

### 2.4 Meta-analysis

Meta-analytical approach was done according to previous studies using a random-effects model [17,20]. Point estimates and their 95% confidence intervals (CIs) for pooled prevalence was calculated for individual studies. The weight of each study corresponds to the size of square, while crossed lines demonstrate the respective CI. The heterogeneity of the studies was investigated by Cochran's test (with a significance level of less than 0.1) and its combination using *I*^2^ statistics (with a significance level greater than 50%) [21]. In case of model heterogeneity, random effects were used by variance image method. The odds ratio (OR) index was used to combine the results of studies. Due to the noticeable heterogeneity of the included studies, meta-regression was utilized as well. The Egger and Begg's test was, also, used to evaluate the publication bias [22]. This kind of bias, if present, skews the results and published reports are not a representative sample of the available evidence anymore. *P*-value less than 0.05 were considered statistically significant. The subgroup analysis was performed based on gender, residence, blood group, Rh, history of blood transfusion, education level, contact with cat, contact with soil, consumption of raw/undercooked meat, raw milk/egg consumption, raw/unwashed vegetable consumption and gardening or agriculture. Power analysis was used to estimate the power of effect sizes. All analyzes were performed by the comprehensive meta-analysis (CMA) statistical software version 2.

In this research, we considered sensitivity analysis to evaluate the impact of each study on the overall findings. Through this method, the overall effect is examined after removing each study to determine any differences with the final result.

### 2.5 Quality assessment

The study quality was evaluated using the Newcastle-Ottawa Scale [[23], [24], [25]], with scoring based on three aspects: Selection (up to five stars), Comparability (up to two stars), and Outcome (up to three stars).

## 3 Results

**Fig. 1:**
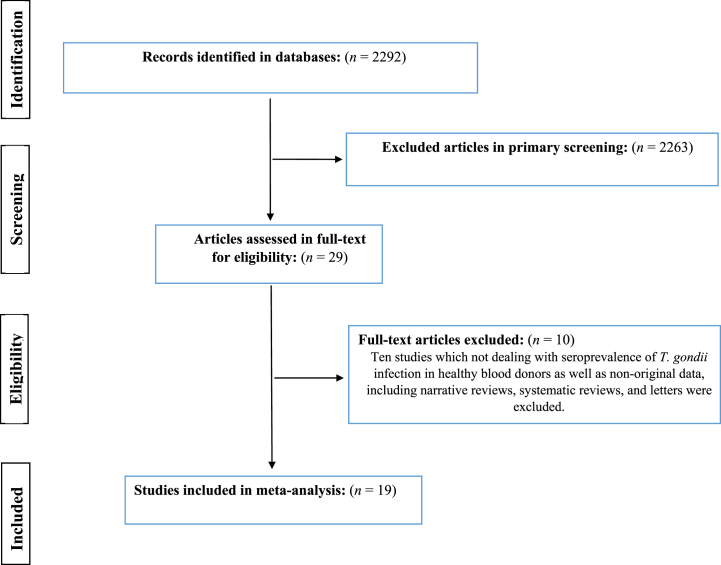
Flowchart showing the search and selection methodology used, which follows the PRISMA guidelines.

**Fig. 2:**
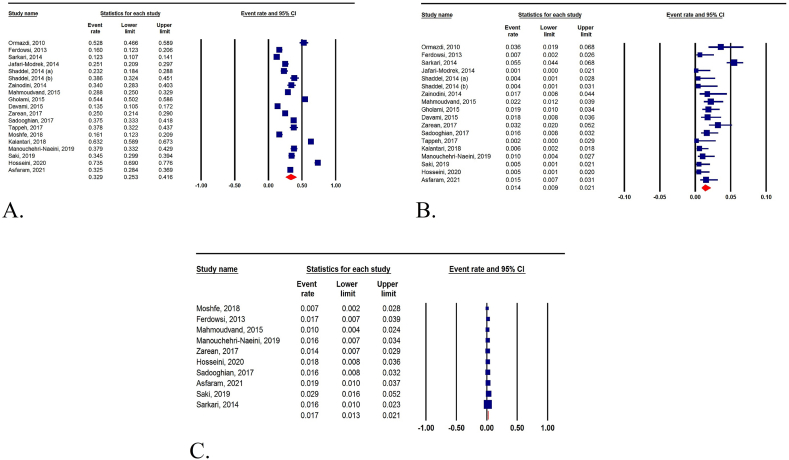
Forest plot diagram of the present systematic review and meta-analysis based on only IgG (A), only IgM (B), and both IgG and IgM antibodies (C) in the Iranian healthy blood donors.

As shown in Fig. 1, we identified 2292 retrieved records in the initial search of databases, and following removing duplicates and/or irrelevant papers, 19 research article were identified eligible to be included in this systematic review and meta-analysis. The Baseline characteristics of included studies in terms of first author's name, publication year, province, method, sample size, and the rate of *T. gondii* exposure are presented in Table 1. Included studies were performed in 12 provinces in Iran (Khorasan Razavi, Tehran, Kerman, Fars, Mazandaran, West Azerbaijan, Sistan and Baluchistan, Hamadan, Kohgiluyeh and Boyer-Ahmad, Chaharmahal and Bakhtiari, Khuzestan, and Ardabil), and most studies were done in Khorasan Razavi and Fars (n = 3 per province), as well as Tehran, Kerman, and Mazandaran (n = 2 per province). Among 19 included studies [[26], [27], [28], [29], [30], [31], [32], [33], [34], [35], [36], [37], [38], [39], [40], [41], [42], [43], [44]], 8226 apparently healthy blood donors were tested regarding *T. gondii*-specific IgG and/or IgM antibodies, among which 32.9% (95% CI: 25.3%–41.6%) were seropositive only for IgG, 1.4% (95% CI: 0.9%–2.1%) were seropositive only for IgM, and 1.7% (95% CI: 1.3%–2.1%) were positive for both IgG and IgM antibodies (Fig. 2A–C).

**Table 1:** Baseline characteristics of included studies.

	First author	Publication year	Province	Method	Sample size	IgG positive	IgM positive	Both IgG/IgM positive	Reference
1	Ormazdi	2010	Tehran	ELISA	250	132	9		[26]
2	Ferdowsi	2013	Razavi Khorasan	ELISA	300	48	2	5	[27]
3	Shaddel	2014	Fars	ELISA	250	58	1		[28]
4	Sarkari	2014	Fars	ELISA	1480	182	81	23	[31]
5	Shaddel	2014	Tehran	ELISA	223	86	1		[29]
6	Zainodini	2014	Kerman	ELISA	235	80	4		[30]
7	Jafari-Modrek	2014	Sistan and Baluchestan	ELISA	375	94	0		[32]
8	Gholami	2015	Hamedan	ELISA	540	294	10		[34]
9	Davami	2015	Fars	ELISA	400	54	7		[35]
10	Mahmoudvand	2015	Kerman	ELISA	500	144	11	5	[33]
11	Tappeh	2017	West Azerbaijan	ELISA	270	102	0		[37]
12	Sadooghian	2017	Razavi Khorasan	ELISA	491	184	8	8	[38]
13	Zarean	2017	Razavi Khorasan	ELISA	500	125	16	7	[36]
14	Moshfe	2018	Kohgiluyeh and Boyer-Ahmad	ELISA	285	46		2	[39]
15	Kalantari	2018	Mazandaran	ELISA	500	316	3		[40]
16	Saki	2019	Khuzestan	ELISA	380	131	2	11	[41]
17	Manouchehri-Naeini	2019	Chaharmahal and Bakhtiari	ELISA	385	146	4	6	[42]
18	Hosseini	2020	Mazandaran	ELISA	400	294	2	7	[44]
19	Asfaram	2021	Ardabil	ELISA	462	150	7	9	[43]

With respect to subgroups, most studies assessed the association between seropositivity and gender (n = 11), blood groups (n = 9), residence (n = 8), education level (n = 8), consumption of raw/undercooked meat (n = 8), and contact with cat (n = 7) (Table 2). Notably, 5162 males and 451 females were assessed in terms of *T. gondii* exposure in 11 studies, among which 1821 [38.3% (95% CI: 28.6%–50%)] and 230 individuals [54.5% (95% CI: 42.6%–65.9%)] were found to be seropositive (OR = 1.98; 95% CI: 1.52–2.58; *P* < 0.001). Among included studies, 488 out of 866 individuals who had a previous contact with cats were seropositive, showing a 56.1% (95% CI: 43%–68.4%) seroprevalence (OR = 2.41; 95% CI: 1.70–3.41; *P* < 0.001). Also, 334 out of 473 individuals with soil contact, were seropositive regarding *T. gondii* exposure with an estimated seroprevalence of 66.9% (95% CI: 52%–79%) (OR = 2.83; 95% CI: 1.07–7.45; *P* = 0.035). Consumption of raw/undercooked meat was examined as a risk factor among 3523 individuals, among which 816 positively answered and 407 of them were *T. gondii* seropositive [48.5% (95% CI: 34.9%–62.4%)] (OR = 1.95; 95% CI: 1.03–3.70; *P* = 0.039). In addition, 1169 out of 1758 individuals consumed raw/unwashed vegetables, among which 688 persons were seropositive [54.2% (9%% CI: 33%–74%)] (OR = 1.70; 95% CI: 1.25–2.31; *P* = 0.001). With respect to other subgroups, higher seroprevalence rates were estimated among individuals in rural areas [45.4% (95% CI: 30.2%–61.5%], with under-diploma level of education [39.8% (95% CI: 27.3%–53.8%], with a gardening history [44.5% (95% CI: 35.1%–54.3%], consuming raw milk/egg [52.9% (95% CI: 38.7%–66.6%], with a history of blood donation [40.5% (95% CI: 24.1%–59.4%)], having B blood group [41.5% (95% CI: 28.2%–56.1%)], and having positive Rh [38% (95% CI: 17.1%–64.6%)]. Nevertheless, no remarkable association was found between a positive *T. gondii* exposure with residence, blood group, Rh, history of blood transfusion, gardening, education level, and raw milk/egg consumption (Table 2).

**Table 2:** Risk factors associated with Iranian apparently healthy blood donors to have *T. gondii* infection according to their serological evidence.

Variables	No. of Studies	No. of blood donors	No. seropositive for *T. gondii*	Pooled seroprevalence (95% CI)	OR (95% CI)	*P*-value	Heterogeneity *I*^2^ (%)	References
***Gender***								
Male	11	5162	1821	38.8 (28.6–50.0)	1			[26,[31], [32], [33],[36], [37], [38],^40^,[42], [43], [44]]
Female		451	230	54.5 (42.6–65.9)	1.98 (1.52–2.58)	< **0.001**	23.7	
***Residence***								
Urban	8	2479	976	38.3 (27.7–50.1)	1			[33,36,[38], [39], [40],[42], [43], [44]]
Rural		1044	500	45.4 (30.2–61.5)	1.37 (0.97–1.93)	0.069	73.8	
***Blood group***								
A	9	1467	510	34.9 (23.9–47.8)	0.92 (0.75–1.14)	0.490	41.9	[[31], [32], [33],[38], [39], [40],[42], [43], [44]]
B		1100	404	41.5 (28.2–56.1)	1.14 (0.81–1.60)	0.430	0.0	
AB		430	167	39.2 (27.6–52.1)	1.07 (0.85–1.36)	0.526	41.9	
O		1870	630	37.7 (25.2–52.2)	1			
***Rh***								
Positive	4	2238	685	38.0 (17.1–64.6)	1.01 (0.78–1.30)	0.938	0.0	[31,32,42,44]
Negative		391	137	37.4 (18.5–61.2)	1			
***Education level***								
Under diploma	8	2485	758	39.8 (27.3–53.8)	1.16 (0.90–1.51)	0.241	66.1	[31,33,36,[38], [39], [40],^42^,^44^]
Diploma and above		2057	836	36.5 (23.5–51.9)	1			
***Contact with cat***								
Yes	7	866	488	56.1 (43.0–68.4)	2.41 (1.70–3.41)	<**0.001**	59.5	[33,36,38,40,[42], [43], [44]]
No		2372	940	37.3 (25.1–51.4)	1			
***Contact with soil***								
Yes	3	473	334	66.9 (52.0–79.0)	2.83 (1.07–7.45)	**0.035**	91.5	[39,40,44]
No		712	324	40.2 (12.8–75.4)	1			
***Consumption of raw/undercooked meat***								
Yes	8	816	407	48.5 (34.9–62.4)	1.95 (1.03–3.70)	**0.039**	88.5	[33,36,[38], [39], [40],[42], [43], [44]]
No		2707	1069	33.3 (21.7–47.3)	1			
***Raw milk/egg consumption***								
Yes	5	454	223	52.9 (38.7–66.6)	1.20 (0.87–1.66)	0.250	40.9	[33,36,40,42,44]
No		1831	839	46.7 (28.5–65.9)	1			
***Raw/unwashed vegetables consumption***								
Yes	4	1196	688	54.2 (33.0–74.0)	1.70 (1.25–2.31)	**0.001**	0.0	[33,40,42,44]
No		589	181	38.7 (21.7–58.9)	1			
***Gardening or agriculture***								
Yes	3	579	246	44.5 (35.1–54.3)	1.89 (0.81–4.40)	0.140	90.8	[33,38,43]
No		874	281	30.5 (20.6–42.7)	1			
***History of blood transfusion***								
Yes	6	71	32	40.5 (24.1–59.4)	1.02 (0.61–1.70)	0.923	0.0	[33,36,[38], [39], [40],^42^]
No		2590	984	39.0 (22.1–51.4)	1			

**Fig. 3:**
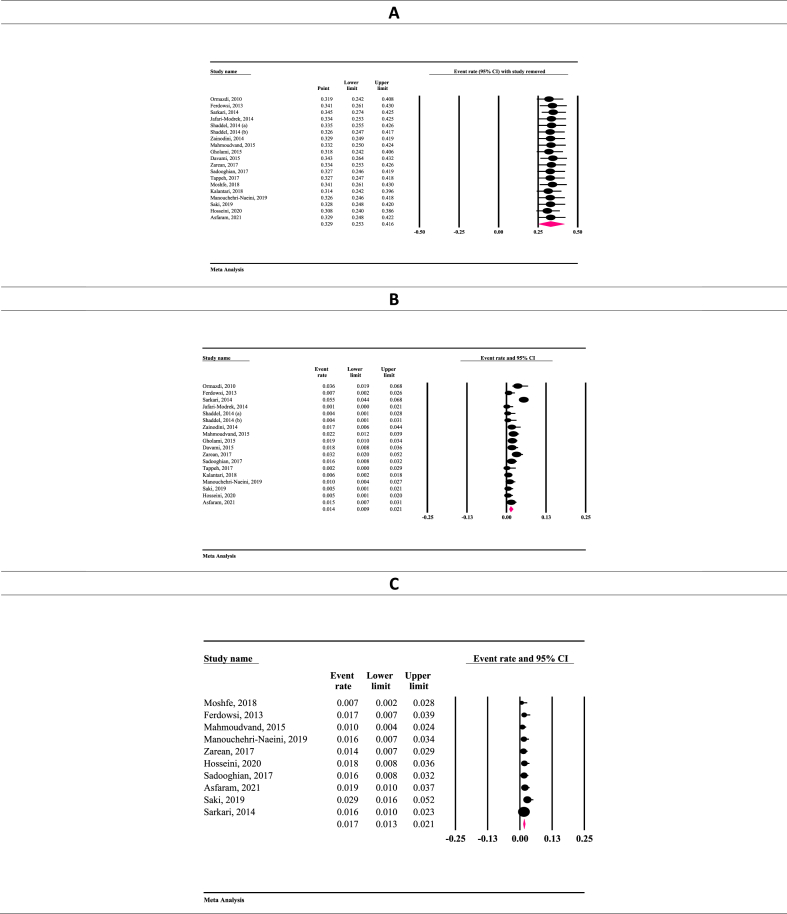
The results of sensitivity analysis based on only IgG (A), only IgM (B), and both IgG and IgM antibodies (C).

**Fig. 4:**
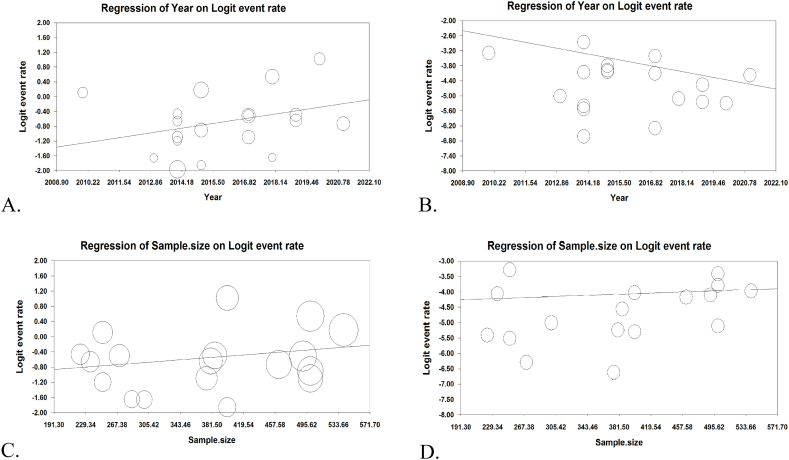
Linear meta-regression analyses of the seroprevalence of *T. gondii* infection in the Iranian healthy blood donors according to time period in terms of IgG (A) and IgM antibodies (B). Meta-regression analyses of the seroprevalence rates of IgG (C) and IgM (D) specific antibodies against *T. gondii* infection based on sample size.

**Fig. 5:**
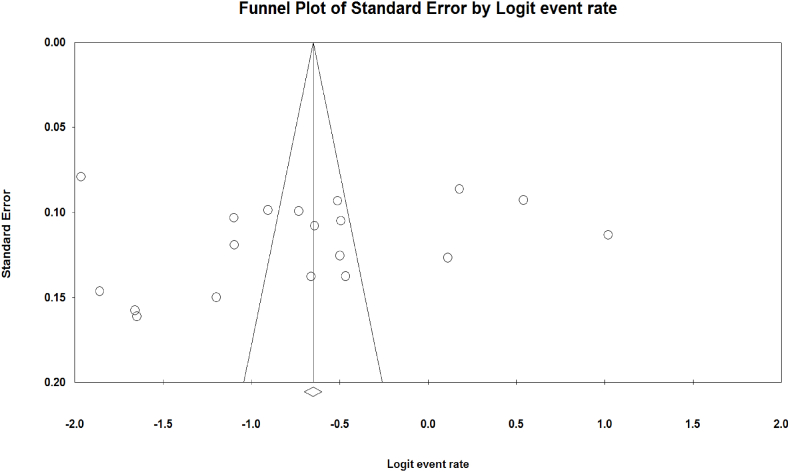
Funnel plot for detecting publication bias.

The result of sensitivity analysis demonstrated that the prevalence remained consistent after removing each article (Fig. 3A–C). Meta-regression results were premised on year of studies and sample sizes. It was shown that *T. gondii* seroprevalence among IgG-positive Iranian blood donors was increased by increasing the year of studies (Reg = 0.097, *P* < 0.001), in contrast to IgM positive donors (Reg = −0.176, *P* < 0.001). Such inconsistency was observed regarding sample size; significant association was found among IgG-positive donors (Reg = 0.001, *P* < 0.001), whereas no significant association was found among sample size and IgM-positive individuals (Reg = 0.0009, P = 0.373) (Fig. 4A–D). The results of the Egger (*P* = 0.575) and Begg test (*P* = 0.151) showed no evidence of publication bias. The funnel plot for detection of publication bias is shown in Fig. 5. Due to the high heterogeneity in the results of meta-analysis, power analysis was used to estimate the power of effect sizes (Table 3).

**Table 3:** The results of power analysis.

Variables	Level of heterogeneity	1- β (Power)
Gender (Male/Female)	No heterogeneity	0.99
Residence (Rural/Urban)	Moderate heterogeneity	0.51
Blood group (A/O)	Low heterogeneity	0.17
Blood group (B/O)	No heterogeneity	0.72
Blood group (AB/O)	Low heterogeneity	0.15
Rh (Positive/Negative)	No heterogeneity	0.11
Education level (Under diploma/Diploma and above	Moderate heterogeneity	0.68
Contact with cat (Yes/No)	Moderate heterogeneity	0.99
Contact with soil (Yes/No)	High heterogeneity	0.94
Consumption of raw/undercooked meat (Yes/No)	High heterogeneity	0.97
Raw milk/egg consumption (Yes/No)	Low heterogeneity	0.78
Raw/unwashed vegetables consumption (Yes/No)	No heterogeneity	0.98
Gardening or agriculture(Yes/No)	High heterogeneity	0.44
History of blood transfusion (Yes/No)	No heterogeneity	0.03

The modified Newcastle–Ottawa Scale assigned scores between 6 and 9 out of a total possible 9 (Supplementary Table 1). Despite inherent limitations in cross-sectional studies, all the included studies demonstrated sufficient quality, placing them in the top third of the scale, making them eligible for inclusion in the current review.

## 4 Discussion

There are substantial challenges in blood transfusion safety, regarding donor selection to post-transfusion surveillance, in most low-middle income countries [45]. Interestingly, TTIs are particularly endemic in low-middle income countries, comprising hepatitis B virus (Asia), human T-cell leukemia virus (Caribbean), malaria and HIV (Sub-Saharan Africa) as well as *T. gondii* infection (Brazil and eastern Africa). Suboptimal testing regarding TTIs is a crucial challenge in these areas [15]. Since existing therapeutics are not adequately effective and no commercial vaccine is available against *T. gondii* infection [[46], [47], [48]], strategies are set to reduce the risk of transmission and subsequent harsh sequelae of toxoplasmosis. It was previously shown that most of the efforts to determine *T. gondii*-infected blood donors were done in Asia, particularly in Iran [16]. Current systematic review and meta-analysis was performed to determine the nationwide rate of *T. gondii* exposure and associated risk factors among Iranian blood donor population.

The present meta-analysis was done using 19 included serological studies published during the last decade, mostly in Khorasan Razavi and Fars provinces (3 studies per province). Most studies had a sample size below 500, with the exception of a multi-center study by Sarkari et al. (2014) [31], where they examined 1480 blood donors in 5 different counties of Fars province. Based on our results, detection of specific anti-*T. gondii* IgG and IgM antibodies in 2666 and 168 individuals out of 8226 examined subjects, respectively, indicates a prevalence of infection of 32.9% and 1.4%. Of note, 83 of 8226 individuals were found to possess detectable specific IgM and IgG antibodies, leading to a 1.7% prevalence of a probably recent infection. Several epidemiological studies have proven that *T. gondii* infection is highly widespread and of utmost significance in Iran [17,49]. A considerable seroprevalence (39.3%) has been estimated for *T. gondii* exposure among the Iranian general population [50]. Also, two meta-analyses reported significantly high seroprevalences among clinically at-risk groups of pregnant women and immunocompromised patients, with 41% (95% CI = 36%–45%) and 50% (95% CI = 43.85%–56.17%), respectively [51,52]. A similar study in mainland China reported a very low IgG seroprevalence [6.26% (95% CI: 4.62%–8.13%)] among 40 relevant records, in comparison with our results [53]. In our study, ELISA was the only screening test regarding *T. gondii* exposure, whereas indirect hemagglutination assay (IHA) was additional diagnostics in Chinese studies; however, it has been shown that no discrepancy presents in sensitivity and specificity of both tests [54]. Reportedly, the sensitive ELISA techniques are routinely used for *T. gondii* serodiagnosis, allowing researchers for quantitative and semi-quantitative antibody measurements. Moreover, they can be readily adopted to screen large populations and remain as inexpensive option in comparison with molecular techniques [55]. Specific IgM antibodies rise during first week post exposure, reach a plateau in a month and decrease thereafter. However, detectable IgM levels, showing active/acute infection, may exist for a year and even longer [56]. In a global perspective, higher IgM seroprevalence were reported from Iraq (5.8%) [57], Thailand (4.3%) [58], Mexico (3.6%) [59], and Ethiopia (2.97%) [60]. IgG seropositivity demonstrates a chronic exposure and higher IgG seroprevalence may indicate the level of exposure of a given population to *T. gondii* infection, which may have occurred through different biological sources. About 1–3 weeks post IgM rise in serum, IgG synthesis begins, reaches a plateau within 2–3 months and remains lifetime after a gradual decrease [56]. As compared with previous studies, higher IgG seroprevalences were documented in Asian (India: 48.6% [61], Jordan: 41.5% [62], Saudi Arabia: 49.3% [63]), African (Ethiopia: 70.29% [60], Sudan: 44% [64], Egypt: 59.6% [65], Kenya: 54% [66]) and American (Brazil: 75% [67], Cuba: 47.5% [68]) countries. Although, *T. gondii* infection is asymptomatic in healthy individuals, it can be fulminant and life-threatening in immunocompromised patients, who highly demand blood transfusion procedures. Nevertheless, more seroprevalence of infection, particularly based on specific IgG, cannot show the risk of transmission of viable parasites by blood transfusion, since specific antibodies persist for several months, and often quite longer, thus their presence long outlasts the narrow parasitemia interval. Heterogeneity among results in studies across the world represents the impact of several factors, including different utilized serological assays with varied cut-off values, geographical and climatic conditions as well as behavioral habits.

It is demonstrated that warm, moist climates in temperate or tropical areas as well as low-altitude regions favor *T. gondii* oocysts to remain infectious for months [69]. On the other hand, durable ultraviolet (UV) rays in hot, dry regions negatively impact the oocyst persistence [70]. This fact was evidenced in two recent studies in blood donors in Mazandaran province, since a considerable part of the examined population was seropositive regarding *T. gondii* exposure [40,44]. This province is bordered between Alborz mountain ranges and the Caspian Sea littoral, resulting in heavy rainfalls and high rate of humidity throughout the year; this climatic milieu benefits oocyst viability and *T. gondii* propagation within different environmental matrices. At a global perspective, Brazil (South America) as well as Ethiopia, Egypt and Kenya (Africa) seemed to possess such environmental benefits for *T. gondii* survival, leading to higher rates of infectivity in blood donors, according to Foroutan-Rad et al. (2016) review [16].

In the current nationwide study, only gender, contact with cat, contact with soil, consumption of raw/undercooked meat and/or raw/unwashed vegetables out of 12 reviewed risk factors were found to be significantly associated with the seropositivity in the Iranian blood donors. Since blood donors are always a portion of general population, those significant risk factors in Daryani et al. study [50] can be merged with ours, highlighting the potency of three well-known risk factors, including contact with cat, raw meat consumption, and raw/unwashed vegetables consumption, which may expose 2.41, 1.95 and 1.70-times higher risk of *T. gondii* infection in blood donors. In Iran, it was previously shown that the weighted prevalence of *Toxoplasma* infection in cats was estimated as 33.6% (95% CI = 22.05%–46.41%) [71]. These parameters seem to be highly influential in parasite dissemination throughout general population of Iran. The educational level was reported to be a significant risk factor for *T. gondii* infection in Chinese blood donors, in contrast to our result. In line with ours, age, occupation, and blood groups were not significantly associated with *Toxoplasma* infection in China [53]. Although, in previous studies from Turkey [72] and Taiwan [73], consumption of raw meat was, also a significant risk factor. But, in several studies no meaningful association was observed between *T. gondii* infecion and this risk factor [52,74,75]. It is worthy to note that transmission of the *T. gondii* through meat consumption depends on the cultural habits of population in a certain region [50,76]. Based on Belluco and colleagues report using meta-analysis approach, 2.6%, 12.3%, and 14.7% of cattle, pigs, and sheep were found positive for *T. gondii* worldwide. The authors confirmed “the role of meat and other meat products as *T. gondii* sources. Increase in consumer knowledge surely influences in reduction of the infection” [76].

Likewise, contact with household cats was a significant, potent contributing factor in blood donors of Mexico [77] and Taiwan [73]. In contrast, contact with cat had no remarkable impact in Serbian [78] and Tunisian [79] blood donors. Based on some significant risk factors reported here, it can be deduced that the dynamics of *T. gondii* transmission in the Iranian blood donors depends, to a great extent, on the ingestion of parasite oocysts through contact with cats, and consumption of raw/unwashed vegetables, and/or *via* ingestion of raw/undercooked meat products of food animals infected with *T. gondii* tissue cysts. A significant portion of blood donors in Iran had chronic/inactive exposure, based on IgG seropositivity and a parallel absence of specific IgM. No IgG avidity tests were done in included studies; however, even low avidity would last longer than the narrow time-frame of parasitemia, and may not truly estimate the status of the infection [80]. Nevertheless, blood transfusion from those a few IgM seropositive individuals to recipients, which are mostly immunocompromised, may relatively elevate the risk of exposure [81]. Despite all these findings, transfusion-transmitted toxoplasmosis (TTT) has not been reported in Iran thus far, and no specific preventive measure has been set neither by the Iranian Blood Bank Organization (IBTO), nor by different international organizations, including the American Association of Blood Banks, Council of Europe (CoE), Caribbean Regional Standards (CRS) and Australian Red Cross (ARC) [82]. Since *T. gondii* can remain alive in refrigerated citrated blood for about 2 months, the Pan American Health Organization (PAHO) recommends *Toxoplasma*-negative leukocyte-filtered preparations of blood products for at-risk groups.

In Iran and many other countries around the world, several studies have detected significant *T. gondii* seroprevalence and emphasized the requirement of a pre-transfusion laboratory testing in order to prevent TTT; however, this may not be applicable for several reasons: **i**) discarding seropositive donors may substantially reduce blood supplies; **ii**) clinically-confirmed cases of TTT have been rare and a routine screening is not performed worldwide; and **iii**) a positive serology does not essentially mean active *T. gondii* infection, and further molecular identification is required.

Our systematic review and meta-analysis met some major limitations: 1) the seroprevalence reports were only available in 12 provinces of Iran, while there is lack of studies in many parts of the country; 2) there was heterogeneity in evaluated risk factors among included studies and a standard questionnaire was lacking; 3) absence of studies trying to detect acute infection by molecular methods; 4) lack of case-control studies to accurately evaluate the status of the parasite exposure; and 5) In light of our limited access to various grey literature sources such as theses, conference papers, unpublished articles, etc,. It is possible that some data may not be fully available for this research project. Therefore, this review has been designed without considering grey literature. Also, IranMedex and Irandoc databases were not searched.

## 5 Conclusion

The present review demonstrated a moderate rate of *T. gondii* exposure among the Iranian blood donors in terms of specific IgG (32.9%), IgM (1.4%) and both IgG/IgM (1.7%) antibodies. Moreover, five risk factors were potently associated with *T. gondii* seropositivity, including gender, contact with cat, contact with soil, consumption of unwashed vegetables and/or undercooked meat. Although *T. gondii* infection is asymptomatic in healthy individuals, it can be fulminant and life-threatening in immunocompromised patients, who are candidates for blood transfusion procedures. However, the seroprevalence data, which are generally the only widely available ones, are not sufficient to estimate the risk of *T. gondii* transmission in a given population, including blood donors. Although, more reliable results can be achieved by utilization of molecular methods in future studies, but for an individual to truly represent a risk of the *T. gondii* transmission, the brief stage of parasitemia, in the very early stages of infection, should be detected, when tachyzoites circulate in the bloodstream and before encystation occurs in destination organs. Accordingly, the cost and effort to perform such tests far outweigh the potential benefits such a distinction could allow. In total, the present systematic review and meta-analysis pooled the serological data from a limited number of Iranian cross-sectional studies to reveal the rate of *T. gondii* exposure among blood donors.

## Data availability statement

Data included in article/supp. material/referenced in article.

## Funding

This research project has been financially supported by Abadan University of Medical Sciences (Grant No. 1333). The funders of the study had no role in study design, data collection, data analysis, data interpretation, or writing of the report. The corresponding authors had full access to all the data in the study and had final responsibility for the decision to submit for publication.

## Ethics approval and consent to participate

This study received the approval from the Abadan University of Medical Sciences Ethical Committee **(**IR.ABADANUMS.REC.1400.090, available at: https://ethics.research.ac.ir/EthicsProposalViewEn.php?id=234724). Ethical issues (including plagiarism, informed consent, misconduct, data fabrication and/or falsification, double publication and/or submission, redundancy, etc.) have been completely observed by the authors.

## CRediT authorship contribution statement

**Masoud Foroutan:** Writing – review & editing, Writing – original draft, Visualization, Validation, Supervision, Project administration, Methodology, Investigation, Funding acquisition, Data curation. **Hamidreza Majidiani:** Writing – original draft, Methodology, Investigation, Data curation. **Soheil Hassanipour:** Visualization, Validation, Software, Formal analysis, Data curation. **Milad Badri:** Writing – review & editing, Visualization.

## Declaration of competing interest

The authors declare that they have no known competing financial interests or personal relationships that could have appeared to influence the work reported in this paper.

## Abbreviations

*T. gondii*: *Toxoplasma gondii*
TTIs: transfusion-transmitted infections
WBC: white blood cell
PRISMA: Preferred Reporting Items for Systematic Reviews and Meta-analyses
ELISA: Enzyme-linked Immunosorbent Assay
IgG: Immunoglobulin G
IgM: Immunoglobulin M
CI: Confidence Interval
OR: odds ratio
CMA: Comprehensive Meta-analysis
IHA: Indirect Haemagglutination assay
TTT: Transfusion-transmitted toxoplasmosis
CoE: Council of Europe
CRS: Caribbean Regional Standards
ARC: Australian Red Cross
PAHO: Pan American Health Organization
IBTO: Iranian Blood Bank Organization
