# Supplementary material for: Toxoplasma gondii seroprevalence in the Iranian blood donors: A systematic review and meta-analysis
Source: Heliyon. 2024 Mar 13;10(6):e28013. doi: 10.1016/j.heliyon.2024.e28013 (PMC10951657; doi:10.1016/j.heliyon.2024.e28013)
Supplement: Multimedia component 1 [file mmc1.docx]

**Supplementary Table 1.** Quality assessment using the Newcastle–Ottawa scale modified for cross sectional studies

| **No.** | **First author** | **Year** | **Selection**  **(maximum of 5 stars)** | **Comparability**  **(maximum of 2 stars)** | Outcome  **(maximum of 3 stars)** | **Total Score** |
| --- | --- | --- | --- | --- | --- | --- |
| 1. | Oormazdi et al. | 2010 | *** | ** | ** | 7 |
| 2. | Ferdowsi et al. | 2013 | *** | ** | *** | 8 |
| 3. | Zainodini et al. | 2014 | *** | * | ** | 6 |
| 4. | Shaddel et al. | 2014 | ** | * | *** | 6 |
| 5. | Sarkari et al. | 2014 | *** | ** | ** | 7 |
| 6. | Modrek et al. | 2014 | *** | * | ** | 6 |
| 7. | Shaddel et al. | 2014 | *** | * | *** | 7 |
| 8. | Davami et al. | 2015 | *** | ** | *** | 8 |
| 9. | Mahmoudvand et al. | 2015 | **** | ** | ** | 8 |
| 10. | Gholami et al. | 2015 | ** | ** | *** | 7 |
| 11. | Hazrati Tappeh et al. | 2017 | *** | * | ** | 6 |
| 12. | Sadooghian et al. | 2017 | **** | ** | *** | 9 |
| 13. | Zarean et al. | 2017 | **** | ** | ** | 8 |
| 14. | Kalantari et al. | 2018 | *** | ** | *** | 8 |
| 15. | Moshfe et al. | 2018 | *** | ** | ** | 7 |
| 16. | Manouchehri Naeiniet al. | 2019 | **** | ** | *** | 9 |
| 17. | Saki et al. | 2019 | *** | ** | ** | 7 |
| 18. | Hosseini et al. | 2020 | **** | ** | *** | 9 |
| 19. | Asfaram et al. | 2021 | *** | * | ** | 6 |

*Indicates one criteria was followed, ** two criteria were followed, ***three criteria were followed, ****four criteria were followed, and ***** five criteria were followed
